# Acute Hydrocephalus, or Tuberculous Meningitis of Children

**Published:** 1840-05-02

**Authors:** 


### MEDICAL EXAMINER.

DEVOTED TO MEDICINE, SURGERY, AND THE COLLATERAL SCIENCES.

No. 18.] PHILADELPHIA, SATURDAY, MAY 2, 1840. [Vol. III.

### BIBLIOGRAPHICAL NOTICE.

### ACUTE HYDROCEPHALUS, OR TUBERCULOUS MENINGITIS OF CHILDREN.

I.—*Dictionnaire de Mèdècine*, Vol. XIX.
II.—*Archives de Générales de Médecine.* September and November, 1839.

In a late number of the Medical Examiner, we noticed the excellent Dictionary of Medicine, now publishing at Paris. In the nineteenth volume is contained an article on Meningitis by Dr. Guersant, which we noticed with commendation, at the same time we stated that, in some respects, it was unjust and exceptionable. In describing the disease, the author has necessarily spoken of the history of the affection, which has been completly misunderstood until quite recently, and, while he alludes to the researches made at the children's hospital by one of the Editors of the Examiner in conjunction with Dr. Rufz, and, in fact, admits that these observations were the first to establish the real character of the disease, he introduces, in the text, the assertion that these observations were not, in reality, original, and if they were original, that another had preceded Drs. Rufz and Gerhard in this matter. The latter remark is the more objectionable, because it is not positively said, but is rather introduced as an inference from the context.

This injustice is of little importance as a purely personal matter, but it is nevertheless natural that some value should be attached to a discovery which, although comparatively limited, was only obtained at the sacrifice of much time and labour; and on the other hand, the general interests of science require that the authorship of a medical discovery should belong to those to whom it is justly due. It is, besides, a matter of undoubted right; and an attempt to take away the credit attached to the discovery of a scientific truth from those to whom it belongs, is quite as great an injustice as any other interference with the right of property. We have been accustomed to respect the character of M. Guersant, and we are not disposed to censure him for an error which we presume is involuntary; nevertheless, it is a matter of duty on our part, to place the subject in its true light, and to sustain the manly and honourable course of Dr. Valleix, the editor of the Paris "Archives," by a simple statement of facts which we shall publish in the next number. The connection of the subject with the history of many important diseases both of infancy and adult life, must be the best apology to our readers, for the length of the papers. The following extract includes the portion of the article, Meningitis, to which we have referred.

"But what is most important to notice, is that this variety of meningitis presents constant alterations and anatomical characters very different from those of ordinary meningitis. Among these, is one which merits particular attention; this is the granulations which are observed in the sub-arachnoidal tissue. Thomas Willis is, perhaps, the first who ever noted this alteration as being very important. He says in his work, De Anima Brutorum, published at Amsterdam in 1682, pars Patholigica, p. 119: '*Nec minus à phlegmone et abcessa, quam ad hujus modi meninginis modis et tuberculalis nonnumqnam cephalalgiæ, lethalis et incurabiles oriuntur.*' This observation of Willis had been forgotten; nevertheless, most authors who have written on hydrocephalus, have spoken of meningeal granulations, but without attaching much importance to them, and without connecting this alteration with the tubercle degeneracy. I have, however, long been struck with this remarkable coincidence, that in meningitis with effusion in the ventricles, we find, together with granulations in the meninges, tubercles, either in the bronchial ganglions, or in the lungs; so that in my clinical lectures, I considered hydrocephalic children as phthisical patients who died by the brain, (considerations surplusieurs maladies des enfans, thesis of M. Leth, Paris, 1839.) I believed, for this reason, that I could already separate meningitis with granulations, from other kinds of cerebral inflammation, and I had assigned it the name of granular meningitis ever since the year 1827, as shown by the registers used in the hospitals during this year, but I had not yet considered, at this period, those granulations to be true tubercles. Later, Dance, in his 'Memoire sur l'hydrociphale,' did not hesitate to approach these granulations to the miliary tubercles found in the pleura and the peritoneum. This truth, which he only announced, has since been placed beyond doubt by the researches of MM. Rufz, W. W. Gerhard, and Constant, who attended, at the same time, the hospital 'Des Enfans Malades.' M. Rufz, who was then attached to the service of my division as '*interne*,' has published in the Archives Génerales de Med. of February, 1833, several observations, in which he points out the granulations. Later, he proved that those granulations, which are met with in most cases of acute hydrocephalus, are of a tubercular nature, by availing himself of new facts in the thesis, which he sustained in 1835, before the Faculty of Medicine. M. Gerhard, (Amer. Journal of Med. Sciences, No.'s for February and May, 1834,) gave a series of observations, made in the 'Hôpital des Enfans,' on cerebral affections, among which, the greater part belong to the disease which now occupies our attention, and which he also considers as a tubercular meningitis. The unfortunate Constant, an exact and conscientious observer, taken away too soon for the science, who was occupied in the same researches as MM. Rufz and Gerhard, and to my knowledge, before them, presented in 1835, together with Dr. Fabre, to the Academy of Sciences, a very good monogragh on tubercular meningitis, which procured them the honour of the Monthyon prize (?) An extract from this monogragh, the source of which the author does not indicate, has been published by Dr. P. N. Green, who had acquired the manuscripts of Constant. This extract, inserted in the Lancet, May, 1836, was afterwards translated in the first volume of the '*Encyclographic des Sciences Medicales*.' An excellent thesis, published in the same year by M. Piet, then an '*interne*' in the 'Hôpital des Enfans,'' contains a just appreciation of the works of those who preceded him, and some observations on tubercular meningitis, peculiar to the author himself. M. Coignet and M. Becquerel, each in a thesis presented to the Faculty of Medicine in 1837 and '38, give a summary under different forms, of the works of their predecessors in tubercular meningitis. Again, M. Le Diberder, in his thesis on the tubercular affection of the pia mater in adults, and M. Valleix, in his paper on the same subject, inserted in the Archives, in the month of January, 1838, establish, in an incontestible manner that the acute hydrocephalus of adults depends on the same tubercular alteration as with children, and presents the same characters.

This pathological truth, appearing now to be well proved, it is then necessary to establish two sections in the history of meningitis. We will give to one the name of *tubercular meningitis*, as being more precise than that of *granular*, we at first adopted, and to the other, that of *simple*, or *non-tubercular* meningitis. This distinction is founded on considerations of pathological anatomy, well determined, and so much more important, that they coincide, as we shall see, with the physiological disorders, often enough to render it possible, in the greater number of cases, to recognise and distinguish these two diseases, which offer, otherwise, as to prognosis and treatment, very different consequences. These two kinds of meningitis have until now been confounded, in nearly all the works that have treated of these subjects. The monograph of MM. Parent and Martinet contains, in particular, nearly twenty observations of tubercular meningitis in children, and nearly as many in adults, so that nearly a third of the observations relate to this disease."

It gives us great pleasure to observe that justice is rendered in this matter by the French journal of greatest authority, we mean the Archives of Medicine. The following remarks are found in the analysis of the Dictionary, published in the number of the Archives for September last, relative to the article Meningitis, by M. Guersant.

"The article Meningitis was vague, and could not be otherwise, when the first edition of the Dictionary appeared; but since then, the exact observation which is now required, has caused this important point of cerebral pathology to be greatly advanced, and it is with the new materials collected within the last few years, that M. Guersant has made the principal additions to the original article. Here we are obliged to enter into some details, because, in relation to the history of the researches on the meningitis of children which have been made at the children's hospital, we cannot agree with the author of the article.

"Every body, until quite lately, attributed to MM. Rufz and Gerhard, the discovery of this form of meningitis, which is so frequent and so fatal in young children. In fact, the former had published in 1833, in the Archives, several observations, in which the existence of tuberculous granulations in the membranes is placed beyond doubt. The latter inserted an analogous memoir in the American Journal (of the Medical Sciences) in 1834, and, finally, M. Rufz presented his thesis, the subject of which is Tuberculous Meningitis, in February, 1835. During all this time nobody disputed their discovery; but lo and behold ! quite recently, M. Fabre in his name as editor, and in that of M. Constant as observer, claims the priority, and sustains that the meningitis was known by them before MM. Rufz and Gerhard had begun their researches. M. Guersant seems to sanction their pretensions in saying, in his article, 'The unfortunate Constant, an exact and conscientious observer, carried off too early for the science, who was occupied with the same researches as MM. Rufz and Gerhard, and to my knowledge before them, presented in 1835, in concert with M. Fabre, to the Academy of Sciences, a very good monograph on tuberculous meningitis, which gained for them the Monthyon prize.' These are very late claims. How can we imagine that an observer who has made his researches before all others, should allow, without complaint, during entire years, the publication of memoirs which carry away the honour of his labours? The fact is so improbable, that we should not hesitate to think M. Guersant was deceived by his recollections, if even we did not positively know, that not only M. Constant had not preceded MM. Rufz and Gerhard in their observations, but that he did not recognise the granulations of the pia mater, until they were pointed out to him by the pupils of the children's hospital, who had all seen them before him. We certainly desire to show all possible respect to the memory of an industrious young physician, but we cannot endure that credit should be given to him for what eminent observers have gained by painful exertion, and we do not doubt that he himself would oppose it, if he were still living. Besides, in questions of priority, nothing should be admitted as testimony but the date of publication, that is, when each has had time and opportunity for complaint, as is the case at present. To admit other principles, would introduce trouble and confusion in literature, and open the way to the most strange pretensions."

To these observations M. Guersant thought it necessary to address the following answer:

To the Editors of the Medical Archives.

*Gentlemen*,—Permit me to claim the insertion in your next number, of the letter which I have the honour to address to you, in order to correct some errors, which have doubtless been overlooked in the hasty preparation of the notice of the nineteenth volume of the Dictionary of Medicine, article Meningitis. Your collaborator makes me say things which I have not said, and passes in silence that which I had explained with sufficient clearness. I am under the greater obligation of addressing this protest (reclamation) to you, as it concerns several of our brethren, both dead and living, and is not confined to myself alone. By a singular preconceived idea, the author of the notice of the article Meningitis, has made the distinction between the two kinds of date from 1833. However, in the short historical sketch which I have given of the progress of the science relatively to this part of the pathology of the brain, I have shown that the origin of the distinction now admitted between the simple and tuberculous meningitis dates back as far as the description of acute hydrocephalus, by Robert Whytt and André de St. Clair. As to the granulations of the pia mater, the discovery of which is attributed by your collaborator to M. Rufz, they were already known by Willis in 1662, as I have proven by a passage of that author which I have cited word for word, and they have been pointed out, and more or less completely described by several of the modern authors who have written on acute hydrocephalus or the meningitis of children. They are well described in an observation of M. Cruveilhier, which is inserted in the work of M. Bricheteau on acute hydrocephalus. The observations VIII, XI, and XIV, of the work of M. Charpentier, contain a good description of it; and our late Colleague, M. Dance, in his memoir on Hydrocephalus, printed in the Archives, says, in speaking of these granulations, that they are of a tuberculous nature, and altogether similar to those met with in the pleura and peritoneum. For myself, I had often ascertained the existence of that kind of alteration at the children's hospital, as the registers of cases which are deposited in the office of administration of hospitals will show, before the observations of the persons alluded to. The register of the year 1824, kept by my son, then my interne, includes several observations of meningitis in which the granulations are very well described; I have pointed them out as one of the pathological alterations met with in meningitis, (Dictionary of Medicine in twenty-five volumes, first edition.) Having observed them more frequently since that period, I had established, in my course of clinical medicine, the distinct nature of granulated or granular meningitis, and I pointed out the fact that children who succumb under this affection present always tubercles in the bronchial glands or the lungs, and are really nothing but phthisical patients, who die of the affection of the liver.

Such was the state of our knowledge in 1833, when M. Rufz published his first observations on acute hydrocephalus, and more recently in 1835, his thesis on the same subject. In giving an account of these observations, of those of M. Gerhard, and of the monograph of MM. Fabre and Constant on tubercular meningitis, I said, *that to my knowledge*, M. Constant was occupied in researches on this subject, and it is a fact well known among those who attended the children's hospital, that M. Constant collected observations on affections of the brain, attended the clinique, and had consequently seen these granulations of the pia mater, which were observed every year, even before M. Rufz was my interne. I have, then, only advanced a fact which is known to all those who frequented the hospital; but I could not attribute to M. Constant, as your coadjutor asserts, the priority of the discovery of granulations in the pia mater. I do not even know that these gentlemen have ever raised a claim which would not, indeed, have a better foundation than that of M. Rufz, since I have carried back the modern discovery of granulations one hundred and fifty years; and hence those granulations of Willis, forgotten for so long a time, were again brought to light by the researches of many physicians and pupils of the hospitals, more than eight years before M. Rufz claimed their discovery for himself. Though I accord to the labours of my young colleagues the merit which is due to them, I have thought that in order to be just to all, I

ought to render up both to the dead and the living what rightly belonged to them.

*M. Valleix's answer to the Reclamation of M. Guersant.*

I regret very much that there should have been a misunderstanding between M. Guersant and myself. I never doubted that M. Guersant had some ideas of the existence of sub-arachnoidal granulations. I even admit, although one might reasonably have some doubts on this subject, that these ideas are traceable as far back as Willis. I, myself, in an article in this journal, cited some observations of Dance, in which this morbid production is described. But, as M. Guersant has well remarked, all of these ideas were entirely insufficient. They were so in this respect; that he, himself, who had studied the question better than any one else, did not hazard his opinion on the value of these lesions, and their relation with the symptoms. We were then compelled to say, that, in some badly determined cases, tubercular granulations were found in the pia mater; but there was a wide distance from these rudiments of knowledge, upon an important pathological question, to an exact description of the symptoms, progress, termination, and characteristic lesions of the disease. And it is right to say, that he who has attained this last end, is really the discoverer.

It is, then, on this part of the subject, that it has appeared to me that the question should be made clear, and I have asked myself, to whom, whether to MM. Rufz and Gerhard, or to M. Constant, belonged the honour of what I believe to be a discovery. Well, I think that if the first researches of M. Constant, which M. Guersant has mentioned in his paper, only led him to the same results as those of his predecessors, and if it was not until after having read the statement of MM. Rufz and Gerhard, that M. Constant saw the accompanying symptoms of a lesion, which he sometimes met without knowing the value of, the constant occurrence of this lesion, the extreme rarity, to say nothing more, of the truly inflammatory meningitis of children, the inflammatory meningitis which was regarded as the most frequent disease before the researches of these two authors, if all of these suppositions are just, and I have the strongest motives for regarding them as such, I believe that I do no one wrong, when I attribute to MM. Rufz and Gerhard the discovery of tubercular meningitis. Before the time of MM. Petit and Serres, ulcerations had been found in the intestines of persons who had died of low forms of fever; the symptoms of these fevers were also known, but no one will hesitate to say that they have rendered us familiar with entero-mesenteric fever; and why? because before them, the relation of constant lesions with certain invariable symptoms, had not been noticed.

I regret having entered so much into detail, but it was my desire to prove that there was no anticipation on my part, and that my intention was most certainly not to throw a veil over the researches of M. Guersant, to which I have always been desirous of rendering justice. If I explained myself badly in my bibliographical article, I hope that this will dispel all uncertainty. My reading on the subject in question, has raised in me the conviction that it was not right to concede the priority to M. Constant; this is all that I wished to say, and I leave to the parties interested, the care of carrying the discussion farther, if they judge it necessary. VALLEIX.

